# Towards large-scale sample annotation in gene expression repositories

**DOI:** 10.1186/1471-2105-10-S9-S9

**Published:** 2009-09-17

**Authors:** Erik Pitzer, Ronilda Lacson, Christian Hinske, Jihoon Kim, Pedro AF Galante, Lucila Ohno-Machado

**Affiliations:** 1 Decision Systems Group, Brigham and Women's Hospital, Boston, MA, USA; 2 Upper Austria University of Applied Sciences, Hagenberg, Austria; 3 Ludwig Institute for Cancer Research, São Paulo Branch, São Paulo, Brazil

## Abstract

**Background:**

Large repositories of biomedical research data are most useful to translational researchers if their data can be aggregated for efficient queries and analyses. However, inconsistent or non-existent annotations describing important sample details such as name of tissue or cell line, histopathological type, and subject characteristics like demographics, treatment, and survival are seldom present in data repositories, making it difficult to aggregate data.

**Results:**

We created a flexible software tool that allows efficient annotation of samples using a controlled vocabulary, and report on its use for the annotation of over 12,500 samples.

**Conclusion:**

While the amount of data is very large and seemingly poorly annotated, a lot of information is still within reach. Consistent tool-based re-annotation enables many new possibilities for large scale interpretation and analyses that would otherwise be impossible.

## Background

Quantitative gene expression experiments provide key information for elucidating biological pathways and understanding diseases. Many methods have been developed over time, from hybridization based, Northern blotting, real-time polymerase chain reaction, high throughput microarrays and serial analysis of gene expression (SAGE), up to modern synthesis based sequencing methods (454, ABI/SOLiD, Illumina/Solexa). Microarrays are a popular technology for large-scale measurement of gene expression. Substantial public repositories have been set up to capture the wealth of information on gene expression generated by researchers world-wide: the National Center for Biotechnology Information's Gene Expression Omnibus (GEO) [1], the European Bioinformatics Institute's ArrayExpress [2] and the DNA Data Bank of Japan's Center for Information Biology Gene Expression Database (CIBEX) [3]. There are, however, several essential pieces of information that are needed to make these measurements useful for anyone other than the original researcher. First, a clear description of the process is necessary to make results reproducible: This includes description of the sample preparation, microarray platform and reporter (probe) sequences. The second essential piece of information is a description of the data processing methods, from the raw image to the expression level. Both steps have been subject to rigorous standardization efforts in the past [4-6]. While it is still challenging to compare measurements, another essential piece of information has received far less attention: Clinical data describing the origin and characteristics of the samples, which is often sparse, inconsistent or simply absent.

Due to the public availability of large microarray repositories and reasonable standardization of measurement values, many 'meta-analyses' and meta-analysis systems emerged [7,8]. These approaches usually merged the lists of differentially expressed genes obtained from previous studies. While this led to emphasis and validation of former results, new insights were less frequent. In order to derive genuinely new results from previous experiments, utilization of *raw measurement values *is almost inevitable.

However, analysing the raw data is challenging, as it requires (1) consistent and high quality probe annotations for all involved platforms, (2) appropriate cross-platform normalization methods, and (3) detailed sample annotations. Only few were able to report success with this approach [9]. While mapping reporter sequences to genes no longer poses a problem, with high quality gene transcripts and several updated re-mappings at hand, normalization is more difficult, as many different factors come into play [10]. Furthermore, sample annotations in popular large collections are hardly structured or consistent across studies and often lack important details.

We addressed the problem of annotating gene expression samples with a consistent set of variables on a large number of existing studies. While this seems to be a daunting task, there were sufficient similarities between samples to make it possible to annotate many samples at once. Moreover, we hypothesized that it was possible to obtain high quality annotations by non-expert individuals who received proper training.

Previous manual or automatic annotation attempts relied or tried to rely on domain experts to do the annotation [11], which was costly and time consuming. However, it has also been shown previously that using students for annotation is a worthwhile alternative to employing experts [12] and that the students themselves quickly become experts for annotating a certain disease. We were confident that students could become adept for the task of annotating a specific set of variables in a specific field.

In the past, several groups proposed annotation tools for microarray experiments [13-18]. These approaches tried to either automatically standardize the existing information or create collaborative platforms together with controlled vocabularies or ontologies to manually create consistent and reusable sample annotations. While there have been some successful cases of automatic term normalization in a smaller scale [11], attempts to automatically curate GEO, the largest public repository, were of limited success, especially when expecting detailed clinical annotation [19]. The large diversity of information and unguided annotation currently present in large repositories call for taking the best ideas from these previous approaches and combining them into a new tool.

## Results and discussion

Our database currently contains the 45 most popular platforms from GEO, 2,445 studies, and 58,432 samples. We also imported a total of 1.6 billion raw measurement values that can be used for new analyses with the help of a cross-platform probe annotation and a cross-platform normalization tool.

We were able to efficiently annotate more than 12,500 samples. More than half of these samples have been redundantly annotated by at least two different annotators. Within four weeks of work of one full time and one part time annotator and a following five weeks of work with four full time annotators, a total of almost half a million variable assignments were made. On average, every sample received 32 annotations.

The most frequently available variables were 'tissue' with 24,602 assignments, followed by 'disease state' with 12,098 annotations, 'sample type' with 9,792 annotations, and 'cell line' with 11,925. Information about genetic modification is also readily available in 11,135 annotations. Other frequently available variables were 'treatment', 'time series', 'gender', 'patient age', 'lymphatic spread', 'estrogen receptor status' and 'tumor type'.

## Conclusion

Many possibilities of further processing are within reach with a method at hand that facilitates an efficient and rich annotation of existing gene expression data. One obvious use case is the regrouping of existing samples to perform new *virtual *studies. Another possible extension is the employment of this or a similar system directly at the public repositories so the uploaded data is annotated by the original submitter.

**Figure 1:**
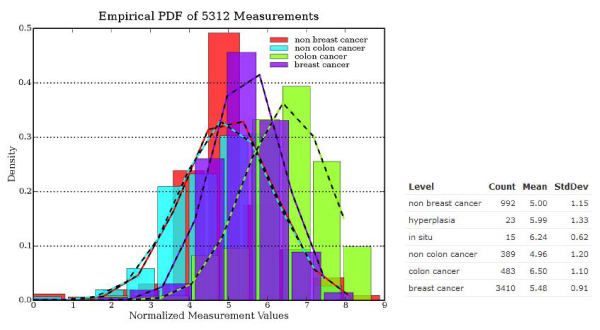
**Screenshot of annotation explorer**. All probes have been re-annotated to the AceView transcript database [22] and normalized using a modified version of quantile normalization [23] that has been adapted for very large datasets. The screenshot shows the distribution of normalized measurement values of BRCA1 for different disease states.

The potential benefit of these detailed annotations is clear: While finding a suitable set of samples across studies was virtually impossible using existing unstructured information alone, we are now able to easily find and compare, for example, 4,405 breast cancer samples with 473 normal breast samples, or 2052 ER+ versus 284 ER- samples. Figure 1 shows a screenshot from the Annotation Explorer interface that uses the annotated and normalized samples to compare gene expression of BRCA1 across several annotated disease states. In a future version, the annotated variables will be made available in an ontology browser to allow for more powerful searching, by expanding the concepts' associations.

We have shown the feasibility of collecting publicly available information about previous microarray experiments and subjecting them to a consistent and efficient annotation. With the current speed and quality of annotation and a total of ten full-time annotators we project the time to annotate all of GEO (currently almost 250,000 samples) with two redundant annotations per sample to be 50 weeks. In the future, we plan to assemble new large datasets and perform new differential expression analyses, avoiding the high cost of sample collection, preparation and hybridization by exploiting existing data.

## Methods

The annotation process started with the identification of possible studies for a certain disease by doing a keyword search. This search yielded a list of studies that had the keyword somewhere in their description or in the description of their samples. Then, annotators proceeded study by study, looking through available information in the local database and following publication links and their respective supplements. After going through all this information, the annotators usually had a good idea of what kind of samples they would subsequently be looking at. Armed with this, they proceeded sample by sample through each study.

Most of the annotation was conducted by one graduate and three senior biology students. An initial pilot annotation was done by one senior biology student and a physician. From this pilot annotation we obtained a feasible set of variables for large scale annotation. In a separate report [20] we provide more details about the quality of the annotation.

### Import and structuring

The first step towards structured annotation was importing large parts of GEO into a relational database by parsing the SOFT files [21]. While these files all have the same format and lexical structure, the individual study and sample annotations contained therein differed greatly in depth and presentation. We developed a tool that makes it easy to import these files. We mapped section names into a consistent scheme and filtered out poorly annotated sections. We found many examples of poor annotation. Some samples merely contained the compulsory organism and source fields but left out other essential information. Around 25% of the imported samples had descriptions shorter than 30 characters, and many just contained words like 'NA', 'null', the organ or a cryptic identifier.

Our database has a straightforward structure and captures just the essentials, like title, description, and organism for sample and studies. This information is the same for all popular expression databases like ArrayExpress or CIBEX, and the importer can be adapted to pull data from these sources.

We created a custom web front-end that showed the most consistently present fields and linked back to the original GEO entries. This web-platform was subsequently extended as a platform for uniform sample annotation. Moreover, we re-annotated probe-to-gene mappings of most of our 45 locally available microarray platforms using AceView [22] as a universal reference. We have created an automatic microarray annotation tool including an up-to-date high quality gene-transcript database that will be published soon.

### Annotation system

One of the major difficulties was finding suitable variables that were frequently available and uniquely iden-tified concepts, while keeping the number of variables low. A concise and complex medical nomenclature had to be clearly separated into distinct terms and predefined choices, enabling non-expert annotators to comprehend existing descriptions and correctly label the described samples.

The underlying idea of the annotation system was to freely attach variables to samples, allowing any variable to be used for any sample. New variables could also be created and assigned if necessary. However, after the initial pilot study, a predefined set of variables with a predefined range of values was established for every disease through discussion and evaluation by experts. This encouraged consistent use of variables and values while at the same providing some freedom to capture seemingly important additional information. To further promote consistency between annotators, newly created variables and non-predefined values were visible to all annotators, albeit less prominently displayed than predefined variables in order to avoid cluttering the interface. This helped us keep a consistent naming scheme even for less important variables in case this information was also available. This guided yet flexible system proved extremely useful as we extended our annotation efforts to cover more diseases.

To facilitate faster annotation of frequently used values, new values could later be added to the set of predefined values and were then directly visible. Predefined variables were grouped into the categories 'essential', 'patient', 'treatment', and 'sample'. Variables were categorized according to individual diseases they appeared in. So far we created these sets of variables and annotated samples for breast cancer, colon cancer, insulin dependent diabetes mellitus, rheumatoid arthritis and systemic lupus erythematosus.

Definition of new variables and possible values is performed using a text format. After review, these are added into the active list of variables by running a script on the text file. This way it is also possible to create new annotation forms that consist of existing variables using the variable definition format shown in Table 1.

**Table 1:** Variable definition file

Colon Cancer:Patient:*age
Colon Cancer:Sample:*genetically modified
Colon Cancer:Sample:*tumor size
Colon Cancer:Essential:tissue, normal, benign, cancerous, non-colon

This example shows the definition of a small annotation form *Colon Cancer *with the two groups *Patient *and *Sample *that contain the variables *age*, *genetically modified *and *tumor size *which reference previously defined variables and one new variable *tissue *with the predefined choices *normal*, *benign*, *cancerous *and *non-colon*.

### Annotation tool

**Figure 2:**
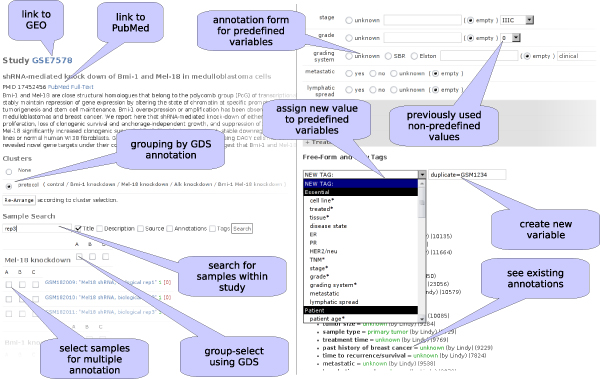
**Screenshot of annotation tool**. *Left: *Several links lead the annotators to additional information. The samples within one study can be searched, grouped and organized to accelerate the annotation of similar samples. *Right: *Predefined variables and values facilitate the annotation. The freedom of defining new variables or new values for existing variables and a way to find them help in capturing as much information as possible in an organized way.

We built a web-based annotation platform to collaboratively create sample annotations in our expression database. Figure 2 shows several useful features of this platform. On the left side, the screenshot shows the study view. All samples and studies are linked back to their original entries within the original repository. Our database contains the titles and summaries of studies and samples which are usually the titles and abstracts of the corresponding publication. The publication itself can be obtained by following a link to the PubMed database. Previous annotations of studies, called GEO datasets (GDS) provided a grouping of samples. Unfortunately, these annotations were available in only 20% of the cases and provide only a group distinction in one variable. Another way for annotators to find similar samples within one study was by searching for sample title, description, source, GDS annotation text or previous annotations in our system. Several samples could be selected for multi-sample annotation. In the multi-sample annotation form (not shown) the selected annotation was performed for all selected samples at once. It contained a summary for each of the samples and the same annotation interface as for single sample annotation. In addition, all annotations from one sample could be copied to another sample within the web application. At the very bottom of the study view page (not shown in the screenshots), a large text area captured any additional useful information the annotators wanted to collect.

The right side of Figure 2 shows the sample annotation interface: After the local information for each sample was displayed (not shown in screenshot), a form with all predefined variables and values prompted for annotation. Variables, that had already been filled out, did not show up again for the same user. Below the initial section with predefined variables and values the annotator could find a powerful free-form annotation tool that allowed users to find and assign any variable that had previously been used, regardless of whether it was originally predefined or not. It also enabled the annotators to create new variables or assign new values to variables that did not allow free text. This feature proved to be useful in the pilot phase of annotation for a new disease, when it was still uncertain what information would be available. Further below, existing annotations were displayed, showing variable name, assigned value, the author of the annotation, and the number of times the variable had been used for every variable assignment. If the annotated value was one of the predefined values for a certain variable, it was highlighted in green.

The internal format of the annotations was a list of tuples of the form (*sample id*, *variable id*, *value*) inside our relational database that can be used to check for concordant annotations, extract samples with certain properties and their associated gene expression values.

## Competing interests

The authors declare that they have no competing interests.

## Authors' contributions

EP, JK, PG and LOM acquired the original data, performed data interpretation and guided the import process. All authors were involved in the system design and conceptualization of the user interface. EP implemented all necessary software systems and prepared the draft of the manuscript. RL, CH, LOM and EP selected an initial set of variables. RL, CH and LOM decided about further inclusion of variables after the initial pilot study. CH participated in the pilot annotation. RL supervised the annotation process and interpreted the quality of the annotation. RL and LOM contributed substantial revisions to the manuscript. PG, JK and CH reviewed the software and provided significant feedback for its further development. All authors read and approved the final manuscript.
